# Acute Appendicitis

**Published:** 1899-12-02

**Authors:** 


### ACUTE APPENDICITIS.

So much has been written of late on the subject of appendicitis, and so many different views have been held in regard to the treatment of the disease, that we receive with considerable satisfaction the judicious summing up of the whole matter which has recently been made by Mr. D'Arcy Power.[1] He points out that an inflammation of the vermiform appendix does not differ pathologically from an inflammation elsewhere. But the appendix being an atrophied portion of the intestinal canal it is not always in a normal condition, even in a person who may otherwise be in perfect health, and thus, when inflammation occurs in it, resolution may be impossible. Moreover, being a part of the intestinal canal, where complex putrefactive changes are continuously going on, there need be small wonder that when an inflammation takes place it is very apt to become infected and to end in ulceration, suppuration, or gangrene. Thus appendicitis may be either simple or infective. The simple form ends in resolution or becomes chronic, forming adhesions or leading to sclerosis of the appendix, which again strongly predisposes to further attacks. The infective inflammation runs a peculiarly deadly course owing to the position of the appendix, for although an abscess may be formed behind the peritoneum, it is much oftener found within it.

So long, then, as the case is "simple," that is, not infective, there is no call for operation. On the other hand, one must always be prepared to operate on the first occurrence of unfavourable signs. Medical treatment then may be adopted so long as the patient seems fairly well; if his pulse is regular, firm, and not too rapid; if his respiration is full and painless; his belly but little distended; and if the stools are normal, or there is only slight constipation. In the treatment of such a case it is better not to use opium in any form, for it hides the tenderness and masks the facial changes, which are the only safe indications of the course which the disease is running. At any rate, if given, it should be administered hypodermically by the medical attendant himself, who should so time his next visit that it may take place when the effect of the drug has passed off. In simple cases, a copious enema of warm soap and water may be given, followed by a teaspoonful of sulphate of magnesia dissolved in a couple of wineglasses of warm water given hourly, the dose being repeated six or eight times until the bowels begin to act. A hot boracic fomentation, or an ice-bag, may be applied to the right iliac region, the choice being determined by the relief which the patient obtains. Bed must be kept until all swelling and all tenderness have left the right iliac region. A non-constipating, pappy diet is essential. Now, as to prognosis. The majority of cases of appendicitis recover, and in a first attack it is said to be three to one against a recurrence. But the affection is a very treacherous one, and during the acute stage one must always be on one's guard lest surgical aid be summoned too late, the patient being visited at least three or four times a day.

When things are going wrong, from the occurrence of infection, a change in the appearance of the patient is one of the first things to cause anxiety; he looks anxious and pinched, without being able to assign any cause; his pulse becomes more rapid, smaller, and softer; the respiration becomes embarrassed; the abdomen is more distended, is more acutely tender, and there is increased thickening in the iliac region. The appearance of the patient, the alteration in the pulse, and the increased abdominal tenderness, especially if it exist without any definite tumour, are the most important signs that an appendicitis is not going its normal course towards recovery, and when these signs occur together it is time to operate with as little delay as possible. We must not, however, imagine that one must not operate until these three signs are met with, for the worst cases are sometimes the most deceptive. It is possible for the physician by his remedies to allay the pain, to stop the vomiting, and to keep the bowels open, while the inflammatory process continues unchecked; and a gangrenous appendix may, as in gangrene elsewhere, lead to diminished pain and fall of temperature. Even a flat abdomen and the passage of flatus does not of necessity negative appendicitis in its worst form. In regard to the operation it must be remembered that, although in itself often a matter of considerable simplicity, it is impossible to tell until the abdomen is opened what one will meet with, so that wherever such a course can be adopted it is well that its performance should be entrusted to those who are experienced in such cases.

[1] Brit. Med. Jour., Nov. 25.

